# Mechanical work accounts for most of the energetic cost in human running

**DOI:** 10.1038/s41598-021-04215-6

**Published:** 2022-01-12

**Authors:** R. C. Riddick, A. D. Kuo

**Affiliations:** 1 Department of Mechanical Engineering, University of Michigan, Ann Arbor, MI 48109 USA; 2 Faculty of Kinesiology & Biomedical Engineering Program, University of Calgary, Calgary, T2N 1N4 AB UK; 3 Centre for Sensorimotor Performance, University of Queensland, Brisbane, QLD 4072 Australia

**Keywords:** Biomechanics, Metabolism, Systems biology, Bioenergetics

## Abstract

The metabolic cost of human running is not well explained, in part because the amount of work performed actively by muscles is largely unknown. Series elastic tissues such as tendon can save energy by performing work passively, but there are few direct measurements of the active versus passive contributions to work in running. There are, however, indirect biomechanical measures that can help estimate the relative contributions to overall metabolic cost. We developed a simple cost estimate for muscle work in humans running (N = 8) at moderate speeds (2.2–4.6 m/s) based on measured joint mechanics and passive dissipation from soft tissue deformations. We found that even if 50% of the work observed at the lower extremity joints is performed passively, active muscle work still accounts for 76% of the net energetic cost. Up to 24% of this cost compensates for the energy lost in soft tissue deformations. The estimated cost of active work may be adjusted based on assumptions of multi-articular energy transfer, elasticity, and muscle efficiency, but even conservative assumptions yield active work costs of at least 60%. Passive elasticity can reduce the active work of running, but muscle work still explains most of the overall energetic cost.

## Introduction

The metabolic cost of human running is not well explained, in part because the work and forces of the muscles are largely unknown. There is little energy dissipated by the environment, and so almost all of the action occurs within a cyclic stride, with equal amounts of positive and negative work by muscles^2–4^, at substantial levels of force and therefore energy cost. Although it is difficult to directly measure this information, there is nevertheless nearly a century of evidence^5^ about important factors such as the energetic cost of work performed by muscle, elastic energy return by tendon, and multi-joint energy transfer by muscle^6–10^. These factors could potentially be combined to synthesize a plausible estimate for how much work muscles perform. This might in turn explain a substantial fraction of the overall energetic cost of running. A first step is to quantify the mechanical work performed by the body, both to redirect the body as it moves across the ground, as well as to move its limbs in relation to its center of mass^11–13^. Muscles expend positive metabolic energy to perform positive and negative work, with efficiencies of about 25% and − 120%, respectively (e.g., ex vivo^5^, for pedaling^9^, and for running up or down steep slopes^8^ where work is largely performed against gravity). The cost of positive work is also supported by the biochemical cost of producing and using ATP for muscle cross bridges to perform work, with a net efficiency (in aerobic conditions, excluding resting metabolism) in the muscles of various animals at about 25%^14^. However, during steady, level human running, work is not readily measurable at the muscles, but rather at the body joints, as with the “inverse dynamics” technique (e.g.,^15^). Joint work does not account for multi-articular muscles, which can appear to perform positive work at one joint and negative work at another, yet actually perform no work^6,16,17^. The estimation of muscle work from joint work therefore depends on the assumed degree of multi-articular energy transfer^6^. Joint work can be used to estimate the work done by muscles only with careful consideration of these mechanisms, and is therefore better suited for giving bounds on muscle work as opposed to precise estimates.

A second issue is elastic energy return. Muscles act in series with elastic tendons, which along with other tissues such as the plantar fascia, can store and return energy passively^18–20^. With some of the work performed on the body due to passive elasticity, running can appear to have high positive work efficiencies of 40%^21–23^ or more. At a comfortable aerobic running speed (2.8–4 m/s) Cavagna and Kaneko^24^ reported an efficiency of 50%. Since the efficiency of positive muscle work is about 25%, these higher efficiencies must be due to the passive return of energy in elastic tissues of the body. In vivo measurements of elastic contributions in the gastrocnemius of a turkey^25^ suggest that tendon could account for about 60% of the observed joint work. But the contribution of elastic tissues to human running has been estimated for a select few tendons under specific types and speeds of locomotion^26–29^, leaving elastic contributions unknown for the majority of muscles and tendons of the body.

Elastic energy return has led to alternative measures that correlate with energy cost. For example, Kram and Taylor^1^ proposed that the cost of running is inversely proportional to the amount of time spent on the ground during each step, scaled by body weight. Referred to here as the KT cost, it presumes that much of the work observed at joints is performed passively by elastic tendon, with muscle largely acting isometrically and at high cost^30,31^. This is largely based on the mass-spring model of running, widely used to suggest that the leg acts purely elastically as it hits the ground^32,33^, with tendons doing most of the work. Indeed, the KT cost correlates well with metabolic cost for a variety of animals at different scales^1^, albeit with differing proportionalities for each case. But its proposed independence from work is also problematic. For example, the KT cost cannot explain the cost of running on an incline^34^, where net work is certainly performed against gravity^8^. Even on level ground, in vivo measurements reveal muscles that do not act isometrically, but perform substantial work^26,27,35^. In addition, soft tissue deformations during running may dissipate substantial mechanical energy^2^, which can only be restored through active muscle work. Thus, work by muscle fascicles is likely still relevant to the overall energetic cost of human running.

**Figure 1:**
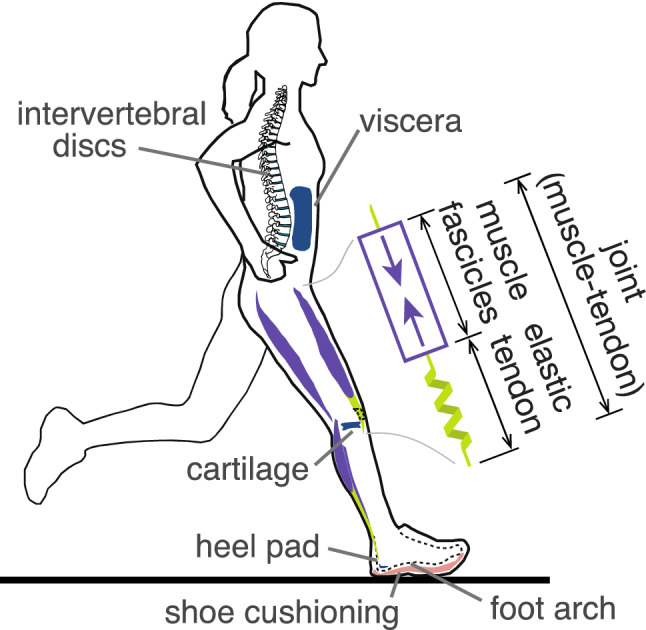
A depiction of the sources of mechanical work in the body during locomotion. Muscle fascicles perform active work in series with passive elastic tendon, and the two together perform work about joints. Soft tissues such as the heel pad and the viscera also deform and dissipate energy over a stride. Passive contributions from series elasticity and deformable soft tissues, along with the structure of multi-articular muscles spanning more than one joint, play an important role in estimating the amount of work performed by muscles.

The present study therefore re-evaluates the contribution of muscle work to running (Fig. 1). This is based on previous estimates for the metabolic cost of work^7,22,34^, but expanded to clarify the upper and lower bounds on each parameter. We account for the effects of multi-articular energy transfer, elastic energy return, and muscle efficiency, and consider how energy dissipation from soft tissues can account for a significant amount of metabolic cost. Recognizing that the assumptions are inexact, our goal is to determine reasonable bounds, rather than an exact estimate, for the cost of work. We then test the degree to which mechanical work can explain the overall energetic cost of running. We hypothesize that even by using the lowest possible bound on the cost of muscle work (taking into account the uncertainty of the model parameters), that muscle work will account for the majority of metabolic cost in running.

## Methods

We estimated the active mechanical work performed by the body during running, and its potential contribution to metabolic cost. We started with joint work measures using standard procedures, supplemented it with recently developed measures of soft tissue dissipation, and then applied simple estimates of multi-articular energy transfer and elastic energy return. Measurement were performed on healthy adult subjects ($N=8$, 7 male, 1 female; 20–34 years) who ran at seven speeds according to each person’s comfort, in randomized order, ranging 2.2–4.6 m/s. Body mass $M$ was 74.9 ± 13.0 kg (mean ± s.d.), and leg length $L$ was 0.94 ± 0.04 m. Subjects ran for a continuous period of 6 min at each speed. This study was approved by the University of Michigan Institutional Review Board and all subjects gave informed consent prior to their participation. All methods and techniques used in the experiment followed the guidelines set forth by the Michigan Institutional Review Board.

**Figure 2:**
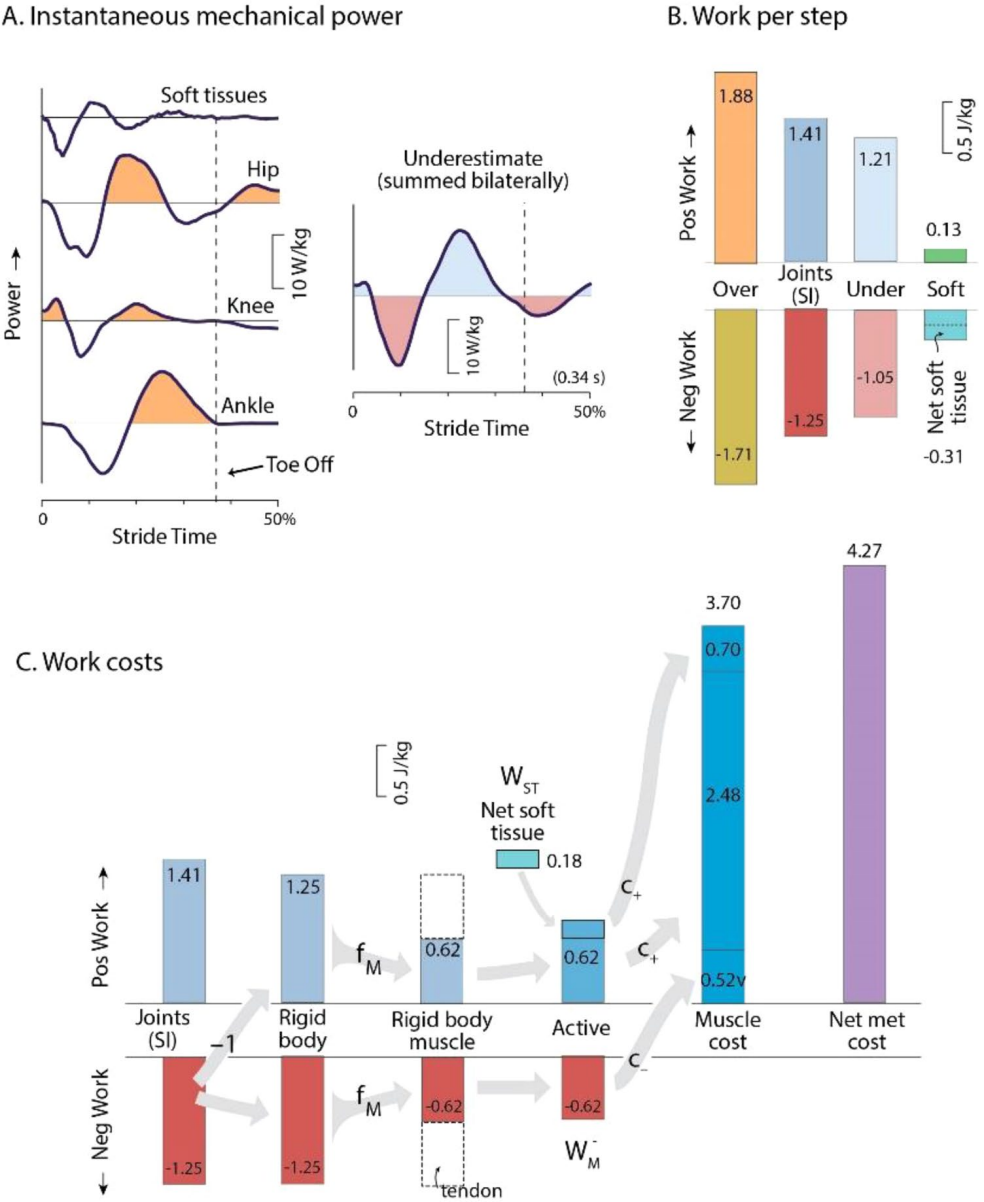
Mechanical work contributions to metabolic energy expenditure, for a representative subject (3.10 m/s, mass = 70.8 kg, leg length = 0.89 m). (**A**) Instantaneous mechanical power of the joints (ankle, knee, and hip), and from soft tissue deformations, over one-half running stride (beginning with heelstrike). Also shown is the summation of all joint powers from both sides of the body, which is an underestimate of power (**B**) Four summary measures of work per step: Overestimate, Estimate (Summed Ipsilateral work), Underestimate, and Soft Tissue work. Positive (negative) work refers to integrated intervals of positive (negative) power. Soft tissue work shown includes positive and negative work per step, and the net (negative, dissipative) work. (**C**) Work costs illustrate metabolic cost contributions. The magnitude of Summed Ipsilateral negative work is treated as an estimate of the joint positive and negative work performed on rigid body segments. This is multiplied by muscle work fraction ${f}_{M}$ (provisionally 0.5) to yield work due to muscle. Active muscle work includes positive work to offset net soft tissue dissipation. Active muscle work is multiplied by the cost of positive and negative muscle work (${c}_{+}$ and ${c}_{-}$) to estimate the energetic cost due to active muscle work.

The kinematic and dynamic data used for this study is the same as presented previously^2^, and briefly summarized here again. Kinematics and ground reaction forces were recorded on a split-belt instrumented treadmill at the University of Michigan. Forces (980 Hz sampling; Bertec, Columbus, OH, USA) and motion capture (480 Hz; PhaseSpace Inc., San Leandro, CA, USA) were collected concurrently, with markers placed bilaterally on the ankle (lateral mallelous), knee (lateral epicondyle), hip (greater trochanter), shoulder (acromion of scapula), elbow (lateral epicondyle of humerus), and wrist (trapezium). Additional tracking markers were placed on the shanks, thighs, trunk, upper arm, lower arm, and upper arm, with three markers on the pelvis (sacrum, left/right anterior superior iliac spine) and two markers on each foot (calcaneus, fifth metatarsal). These data were collected for at least 1 min per trial, with force data filtered at 25 Hz and marker motion at 10 Hz (second-order low-pass Butterworth), and then applied to inverse dynamics calculations (Fig. 2) using standard commercial software (Visual3D, C-Motion, Germantown, MD, USA).

These data were used to compute two kinds of mechanical work. The first was standard rigid-body joint powers, as the work per time needed to rotate and translate (via joint torque and intersegmental reaction forces, respectively) two connected segments relative to each other. We used the so-called 6-D joint power, considered robust to errors such as in joint center locations^13,36,37^.

The second quantity was the dissipative work performed by soft tissue deformations. Briefly, this is the difference between rigid-body joint power and the total mechanical work^2,13,24,38^. The total mechanical work is defined as the rate of work performed on the COM (evaluated using ground reaction forces with no rigid-body assumptions^12^) plus the rate of work performed to move rigid-body segments relative to the COM. In running, this quantity is similar in magnitude to the difference between the positive and negative joint work over a stride^2^, which itself implies that rigid body work does not capture all of the work of running. The term “total mechanical work” is defined as the summation of soft tissue and joint work.

Metabolic cost was estimated through respirometry (Oxycon; CareFusion Inc., San Diego, CA). Both O_2_ consumption and CO_2_ production were recorded on a breath by breath basis and averaged over the final three minutes of each 6-min trial, and converted to gross metabolic rate (in W). Net metabolic rate was found by subtracting each subject’s cost for standing quietly, collected before running. The subjects’ respiratory exchange ratio (RER) was measured to be 0.85 ± 0.09 across subjects, with each individual trial having an average RER of less than 1, indicating mostly aerobic conditions.

### Mechanical work and energy transfer by muscle–tendon

The work performed by joints and soft tissue deformation was used to estimate that done by the series combination of muscle and tendon. To illustrate energy transfer assumptions, we initially consider two opposing sets of assumptions—an Overestimate and an Underestimate—before introducing our intermediate measure. The Overestimate assumes no multiarticular energy transfer between joints, as if all muscles acted uniarticularly. Positive work is thus evaluated by integrating the positive intervals of each joint’s power over a stride (Fig. 2A), and then summing across all joints in both sides of the body, as if they were independent joints (IJ). Multiplying by stride frequency then yields the average rate of positive independent-joint work, ${\dot{W}}_{\text{IJ}}^{+}$. We consider this quantity to be an Overestimate because it disregards energy transfer by multi-articular muscle.

The Underestimate of work takes the opposite extreme, and assumes that simultaneous positive and negative work always cancel each other. This entails summing the powers from all the body joints at each instance in time, yielding summed joint power^13^, and then integrating the positive summed joint power over a stride. Multiplying by stride frequency yields the average rate of positive summed-bilateral (SB) joint work, ${\dot{W}}_{\text{SB}}^{+}$ (Fig. 2B). This is considered an Underestimate of actual muscle–tendon work, because it assumes energy transfer can occur between any two joints, regardless of whether a muscle crosses those joints. The Over- and Under-estimates, ${\dot{W}}_{\text{IJ}}^{+}$ and ${\dot{W}}_{\text{SB}}^{+}$, are roughly analogous to the terms “no between-segment transfer” and “total transfer between all segments” of Williams and Cavanagh^7^, except applied here to transfer between joints rather than body segments.

We introduce our own intermediate muscle–tendon work estimate, termed Summed Ipsilateral (SI) work. It assumes full energy transfer across the joints on each side of the body, but not between the two sides. This has previously been justified based on inter-segmental energetic analysis^39^. This is mostly because there are no muscles that cross the legs and could transfer negative work from one leg into positive work at the other. The average rate of work ${\dot{W}}_{\text{SI}}^{+}$ entails summing the joint powers on one side of the body at each point in time, integrating the positive intervals of this power (Fig. 2B), and then multiplying by step frequency. Of course, further examination of musculoskeletal geometry, neural activation patterns, and loading conditions could yield more intricate estimates of muscle–tendon work. But without full knowledge of individual muscle forces and displacements, we use the Summed Ipsilateral estimate as a simple and not unreasonable set of assumptions, between the aforementioned extremes.

### Metabolic cost of muscle work

We define two quantitative parameters to link muscle–tendon mechanical work to energy expenditure. The first is the proportion of work performed actively by muscle vs. passively by tendon, and second is the metabolic cost at which the active work is performed. The proportion is defined as ${f}_{m}$, the fraction (ranging 0–1) of muscle–tendon work performed by muscle fascicles, such that

1 $${W}_{\text{M}}^{+}={f}_{\text{M}}{W}_{\text{MT}}^{+}$$

where ${W}_{\text{M}}^{+}$ is the positive work of muscle fascicles and ${W}_{\text{MT}}^{+}$ is the positive work of muscle–tendon (applying the proposed Summed Ipsilateral measure, or the Over- or Under-estimate assumptions), and analogously for negative work. In vivo measurements suggest a variety of possible values for ${f}_{m}$, for example 0.40 for turkey gastrocnemius^25^, and 0.26–0.56 for two muscles of running dogs^40^. For humans, cadaver data suggest 0.52 for the Achilles tendon and foot arch^19^. Other indirect data suggest a range of 0.4–0.625^22,24^, depending on energy transfer assumptions. The correct value is unknown, and almost certainly varies with muscle group, loading conditions, and speed. We use a single parameter ${f}_{m}$ to summarize an overall effect for all muscles, and adopt a provisional value of 0.5, while allowing for other possible values (see Table 1).

**Table 1:** The cost coefficient represents how much metabolic energy a unit of mechanical work costs.

	Positive work cost ${c}_{+}$	Negative work cost ${c}_{-}$	Net work cost ${c}_{\pm }={c}_{+}-{c}_{-}$	Muscle work fraction ${f}_{\text{M}}$	Cost coefficient ${f}_{\text{M}}{c}_{\pm }$
Upper bound	4.00^8,9^	− 0.83^8,9^	4.83	0.65^21^	3.14
Lower bound	4.00^8,9^	0	4.00	0.38^22^	1.52

The cost coefficient is calculated by taking into account the amount of work performed by tendon relative to muscle, and the efficiency of positive and negative muscle work. A range of cost coefficients between 1.8 and 3.2 were found by consulting experimental data from the literature.

We characterize the metabolic cost of muscle work with separate parameters for positive and negative work. The positive work cost ${c}_{+}$ is defined as the metabolic energy cost of producing a unit of active positive work, equivalent to the inverse efficiency of pure positive work. An analogous cost ${c}_{-}$ is defined for the metabolic cost of negative work. We adopt provisional values for ${c}_{+}$ and $c\_$ of 4.00 and − 0.83, respectively, equivalent to efficiencies of 25% and $-120\%$^41^, again allowing a range for $c\_$ (see Table 1).

The overall energetic cost of this work ${E}_{\text{work}}$ is summed for rigid body and soft tissue contributions (graphically depicted in Fig. 2C). Soft tissues dissipate net energy (yielding negative ${\dot{W}}_{\text{ST}}$), and muscles must actively perform net positive work to compensate for those losses. The positive cost of making up for such dissipation is therefore ${c}_{+}\left|{W}_{\text{ST}}\right|$. The cost of rigid body work is estimated from the magnitude of negative work from inverse dynamics $\left|{W}_{\text{M}}^{-}\right|$, multiplied by the costs for both positive and negative work. These summed contributions yield

2 $${E}_{\text{work}}=\left({c}_{+}+{c}_{-}\right)\left|{\dot{W}}_{\text{M}}^{-}\right|+{c}_{+}\left|{\dot{W}}_{\text{ST}}\right|.$$

This energetic cost per stride is then multiplied by stride frequency to yield metabolic power ${\dot{E}}_{\text{work}}$ due to active work.

To account for differences in subject size^42^, data were non-dimensionalized using body mass $M$ leg length $L$, and gravitational acceleration $g$ as base variables. Mean power and work normalization constants were $M{g}^{3/2}{L}^{1/2}=2184 \text{W}$ and $MgL=678 \text{J}$, respectively. The mean running speed normalization constant was ${g}^{1/2}{L}^{1/2}=3.04$ m/s. All averaging and statistical tests were performed with dimensionless quantities. In figures, data were plotted with dimensional scales in SI units, using the mean normalization constants.

Statistical tests were performed as follows. We used a linear least-squares fit to relate running speed to mechanical or metabolic rates, and then used Eq. (2) to estimate the metabolic cost attributable to work. We also used the linear least-squares fit to test how other work measures and the KT cost are related to metabolic rate. All regressions were performed allowing each subject an individual constant offset, while constraining them all to a single linear coefficient. The relationship between the predictor and response variables were considered significant when p < 0.05 for the *F-*statistic. Measures are reported in the form *Y* ± C.I. for $\alpha =0.05$ where *Y* is the predicted response of the linear regression model.

## Results

**Figure 3:**
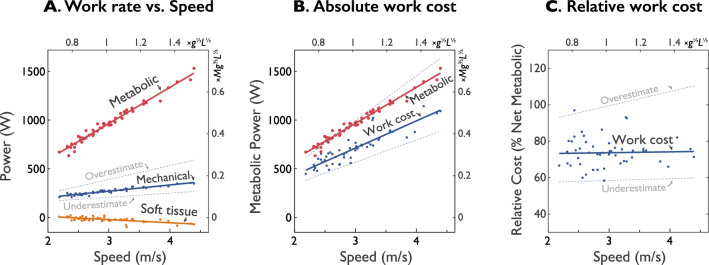
Mechanical work and estimates of absolute and relative metabolic cost vs. speed ($N=8$). (**A**) Average positive work rates: Mechanical work (using Summed Ipsilateral estimate), net Metabolic rate, and net Soft tissue work rate. Also shown are Over- and Under-estimates of work (dashed gray lines) assuming no work transferred between joints by multiarticular muscles, and full transfer, respectively. (**B**) Estimated metabolic power for mechanical work, based on each work rate, along with soft tissue deformations, muscle work fraction, and muscle work cost. (**C**) Relative metabolic cost for mechanical work, showing each cost as a fraction of net metabolic rate. Axes shown include dimensional units, as well as dimensionless units (top and right-hand axes) using body mass, leg length, and gravitational acceleration as base units.

We found that all measures of mechanical work rate and metabolic rate exhibited typical and fairly linear increases with running speed. Mechanical work data are summarized here, with more comprehensive measures reported previously^2^. In terms of standard joint powers (Fig. 2A, representative data), the ankle, knee, and hip powers far exceeded that for the upper body. Soft tissues produced power similar to a damped oscillation (reported previously^2^), and the Over- and Under-estimates of power bracketed the intermediate estimate, as expected. This was also true for the overall Over- and Under-estimates of positive and negative work per stride (Fig. 2B); soft tissues produced net negative work. These observations were consistent across the range of running speeds measured (Fig. 3). As expected, the proposed Summed Ipsilateral work rate increased with running speed (Fig. 3A), and was between the expected Overestimate and Underestimate. Net soft tissue work rates were negative and increased in magnitude with speed. The regression coefficients and statistical outcomes for the relationship between these measures of power and running speed can be found in Table 2.

**Table 2:** Linear relationships between measurements of power (metabolic and mechanical) vs running speed.

Measurements of power	Slope ± 95% CI	Offset	r^2^	*p*
Net metabolic	0.48 ± 0.033	− 0.01	0.98	2E − 31
Summed ipsilateral	0.08 ± 0.011	0.05	0.96	2E − 25
Summed bilateral (underestimate)	0.09 ± 0.014	0.03	0.93	3E − 21
Independent joint (overestimate)	0.19 ± 0.0098	0.01	0.99	3E − 38
Soft tissue	− 0.04 ± 0.020	0.03	0.80	4E − 12

The slope and offset from the linear regression (in dimensionless units) are reported, along with *r*^2^.

The estimated metabolic cost for performing that work was substantial. Applying elastic contributions, the metabolic cost for performing active work (Eqs. 1 and 2) ranged about 500–1000 W over the speeds examined, compared to an overall net metabolic rate of 700–1500 W (Fig. 3B). In relative terms (Fig. 2C), work accounted for about 76% of net metabolic rate (Fig. 3C), with little dependence on running speed (slope = 0.10% per 1 m/s change in speed). In contrast, the Overestimate of work yielded a much higher proportion (slope = 7.1% per 1 m/s change in speed), of 106% at 3 m/s, and actually exceeding 100% of net metabolic rate at most speeds considered. The Underestimate yielded a fairly constant proportions of about 61% (slope = 0.62% per 1 m/s change in speed).

**Figure 4:**
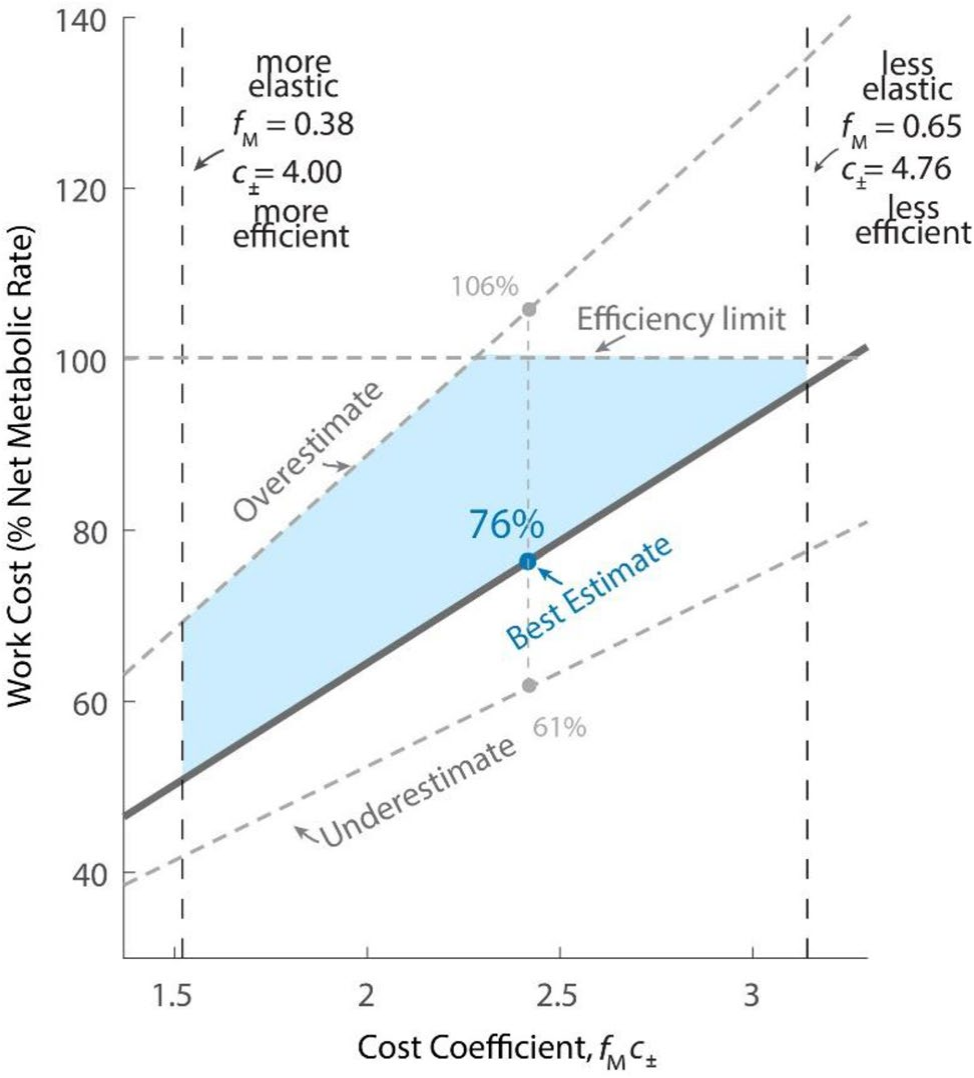
Average work cost as a function of cost coefficient for running at 3 m/s. Relative work cost is estimated metabolic cost of mechanical work divided by overall net metabolic cost. Cost coefficient is defined as fraction of work attributable to muscle from overall muscle–tendon work, multiplied by cost of active work ${c}_{\pm }$. Boundaries are shown for extreme assumptions. Overestimate is for Independent Joints assumption, where muscles only act uniarticularly; underestimate is for Summed Bilateral joint assumption, where work can be transferred from one side of the body to the other. Left and right boundaries are for extremes in muscle work fraction, 38% and 65%, respectively, with constant cost of work. The proposed work estimate (Summed Ipsilateral joints), along with a muscle fraction of 50%, yields 76% of the metabolic cost of running is attributable to active work by muscle. For the same parameters, the Underestimate yields 61% and the Overestimate 106%.

These results are next illustrated as a function of parameters, to facilitate evaluation of assumptions (Fig. 4). Here we use an overall cost for combined positive and negative work, ${c}_{\pm }={c}_{+}-{c}_{-}$, with nominal value 4.83. This is nominally paired with muscle work fraction ${f}_{m}$ of 50%. With these values, the proportion of metabolic cost explained by work was 61% for the Underestimate, 76% for Summed Ipsilateral, and 106% for Overestimate, respectively, across the observed running speeds. Here we also examine two extremes for alternative assumptions. One is to assume a considerably lower fraction of muscle work, ${f}_{m}=0.38$, which would yield a lower fraction of metabolic cost explained, of 43%. On the other hand, assuming that muscle performs more work, ${f}_{m}=0.65$, yields an unrealistic explained amount of 135% (Fig. 4).

Using the nominal efficiency of ${c}_{\pm }$ along with the Summed Ipsilateral cost for work, active work to compensate for soft tissue dissipation accounted for an increasingly larger proportion of the metabolic cost due to work. At the nominal speed of 3 m/s, soft tissue compensation increased the metabolic cost due to work (as predicted by the linear regression) by 23.3%, from 3.00 to 3.70 J/kg. Whereas at the highest speed of 4.6 m/s, soft tissue compensation increased the estimate of cost due to work by 31.5%, from 3.82 to 5.03 J/kg.

## Discussion

We had sought to re-evaluate the degree to which mechanical work performed by muscle can explained the net metabolic cost of running. We considered three sets of assumptions to translate joint work estimates into metabolic cost: how energy is transferred between joints by muscle, how much work is performed passively by tendon, and how much metabolic energy is expended to perform muscle work. Using nominal assumptions for muscle vs. tendon work and muscle efficiency from the literature, we found that about 76% of the metabolic cost of running is attributable to muscle work. We next discuss how our estimates may be interpreted, and how they could be affected by alternate assumptions.

One contributor to the high work cost is dissipation by soft tissues. The dissipation is not typically measured in inverse dynamics analysis, nor incorporated into estimates of metabolic cost. In a typical inverse dynamics analysis, the only work is performed about joints acting between rigid segments, leading to an imbalance of work^2,43^, with more positive than negative work. In fact, soft tissue deformation largely explains this joint work discrepancy^2^. For example, (representative subject, Fig. 2), soft tissues dissipated 0.18 J/kg, explaining much of the positive/negative work discrepancy of 0.16 J/kg at 3.1 m/s. Active work to make up for this dissipation accounted for 0.7 J/kg (16%) of the entire 4.27 J/kg of the net metabolic rate. And at faster speed of 4.6 m/s, that fraction increases to about 31%. Faster speeds entail higher impact between leg and ground, and more energy dissipation. The work to compensate for soft tissue energy dissipation costs substantial metabolic energy.

Another contributor is active work in tandem with passive elasticity. Series elasticity is recognized to perform substantial work passively, and thus to play an important role in running energetics. But even with passive elasticity, our results suggest that the remaining work attributable to muscle accounts for much of the overall energetic cost. This is based on an assumed muscle work fraction ${f}_{m}$, provisionally set to a nominal value of 50%, for which far different values might be appropriate. For example, the plantaris and gastrocnemius of hopping wallabies have a range from only 3–8%^44^. In human, the Achilles tendon appears to facilitate a low muscle work fraction^23,35^. However, many other muscles also participate in running, not all under conditions ideal for tendon elastic work. It is therefore helpful to use the parameter study (Fig. 4) to evaluate other candidate assumptions that lie between these two extremes.

Another factor in our energy estimate is the energetic cost of muscle work. This is mainly for positive work, and is attributable to crossbridge cycling^45^. Thermodynamic principles dictate that this cost likely exceeds ${c}_{+}=4$ (or efficiency does not exceed 25%), due to the biochemical costs of ATP production and for the work of crossbridge cycling^46^. We did not include other effects such as frictional work^47^, muscle co-contraction, isometric force production, or calcium pumping^48^, which would generally be expected to cost energy, and could be lumped into the remaining fraction of energy cost (24%) not explained by fascicle work. We also assumed a small but positive energetic cost to negative work. An extreme assumption would be zero cost for negative work, which would reduce the estimated metabolic cost for work from 76 to about 63%, still a majority of overall metabolic cost.

We also examined alternative assumptions for energy transfer by multi-articular muscles. Although generally unknown in humans, measurement of muscle forces in cat locomotion show significant energy transfer from the ankle to the knee during collision, and from the knee to the ankle during push-off^49^. We therefore consider it unrealistic to assume no such transfer in humans, hence the label of Overestimate for the individual joints (IJ) estimate of work. Indeed, the IJ estimate would yield an entirely unrealistic apparent mechanical efficiency of 102% for running at 3 m/s (Fig. 4). On the other hand, the Underestimate is too low since it assumes that negative work at any joint could be transferred perfectly to positive work at any other joint in the body. We have therefore presented the Summed Ipsilateral (SI) assumption as a better, yet likely low, estimate for work performed by muscles. This model of energy transfer was previously proposed by Willems et al.^39^, although without including the contributions of soft tissues, which we have found important for metabolic cost. It has long been recognized that energy transfer can occur between joints of an individual leg^6,49–51^. Our own estimates summarize the bounds from the possible assumptions and could be improved with more direct muscle measurements from humans.

Our findings could inform other estimates of mechanical work. Others have used independent joint work to evaluate apparent efficiency during locomotion^23,52^, for example yielding unusually high running efficiencies of 35–40%^23^, which they largely attributed to series elasticity at the ankle. But we also believe some of their observed work may be an Overestimate, due to multi-articular energy transfer. Our preferred estimate using summed ipsilateral joint work is more similar to the segmental energy transfer approach of Williams and Cavanagh^7^, except using work at joints rather than between segments, and including soft tissue work not been previously considered. This facilitates estimation of metabolic cost contributions (Eq. 2) with only two main parameters (${f}_{\text{M}}$ and ${c}_{\pm }$) lumped into the cost coefficient. We anticipate that further measurements of muscle and tendon action in vivo will inform better estimates of cost contributions such as work.

**Figure 5:**
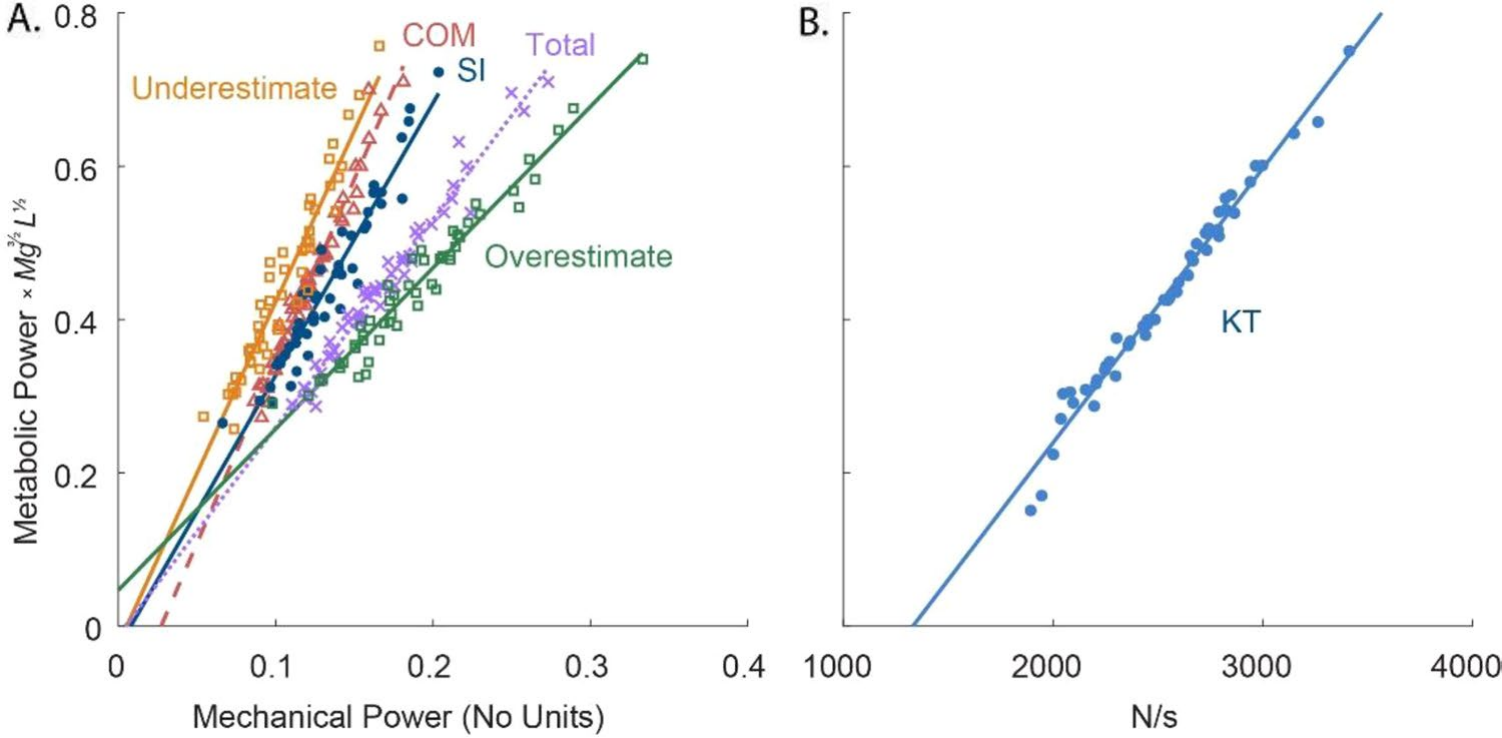
Sample correlates of metabolic cost. (**A**) Correlates: Summed Ipsilateral (SI) work, positive COM work rate, Total mechanical work, Underestimate of joint work (assumes full energy transfer), and the Overestimate of joint work (assumes joint independence). (**B**) The KT measure of body weight divided by ground contact time (Kram and Taylor^29^) compared to metabolic cost. All measures correlate well (r^2^ > 0.9) with metabolic cost. Power is plotted in terms of normalization units, $M{g}^{3/2}{L}^{1/2}$.

There are certainly other costs for running, not attributable to work. Examples include a cost for producing force in the absence or regardless of mechanical work^1,30,31^, or due to the rate at which force is generated^53^. We evaluated the KT cost (Fig. 5) proportional to body weight divided by ground contact time^1^, which correlates quite well with metabolic cost. But several measures, including various estimates of work, also correlate well (Fig. 5, Table 3). We consider it more mechanistic for a cost to depend on applied muscle force or work, rather than general parameters such as body weight. For example, “Groucho running” on flexed knees^54^ costs 50% more energy than normal running, whereas the KT cost would predict a decrease, due to increased ground contact time. We suspect that the high cost of Groucho running is due to greater muscle forces and work with flexed knees^55^ even though body weight remains unchanged. Furthermore, reinterpretation of the KT cost reveals that it could be equivalent to a cost of performing mechanical work under appropriate assumptions (see Supplementary Appendix S1). Work is certainly needed to accelerate during running, or to ascend an incline, and it appears to account for a majority of the cost for level ground. We do acknowledge other costs, potentially for isometric force production, but mainly for the 24% of energy not explained by work.

**Table 3:** Linear relationships between running measurements and metabolic cost.

Measurement	Slope ± 95% CI	Offset	r^2^	*p*
Estimate: summed ipsilateral (SI)	3.55 ± 0.50	− 0.03	0.92	7E − 20
Overestimate: individual joints (IJ)	2.10 ± 0.20	0.06	0.96	9E − 26
Underestimate: summed bilateral (SB)	4.45 ± 0.73	− 0.02	0.90	7E − 18
COM work rate	4.75 ± 0.40	− 0.13	0.97	7E − 28
Total mechanical work rate	2.71 ± 0.23	− 0.01	0.97	1E − 27
Kram and Taylor^1^ (KT) cost	3.58E − 4 ± 3.1E − 5	− 0.48	0.97	7E − 28

The slope and offset from the linear regression (in dimensionless units) are reported, along with *r*^2^. Each measurement is a measure of mechanical work performed at the joints or on the COM.

The present study has a number of limitations. Our results are specific to humans running at a limited range of speeds, and it remains to be seen how well work can explain energy cost over a wider range of speeds. In particular, work may be less explanatory for other animals, particularly smaller ones where muscles are turned on or off more quickly^56^. Such force cycling costs may be applicable to humans as well^53,57,58^. And our model for the cost of mechanical work could be applied to other activities such as walking^43,59^ and hopping, not considered here. Similar assumptions for the cost of work in incline running reported by Minetti et al.^34^ (without accounting for soft tissue deformations) suggest that this approach could be applicable beyond level-ground running. The present model for estimating metabolic cost is mostly based on motion data, whereas a more comprehensive and mechanistic model would include body dynamics and predict both motion and energy cost.

But the primary limitation is in the cost coefficient, which attempts to aggregate information from empirical data. Better estimates could be obtained as in vivo measurements of muscle state (e.g. ultrasound^60^) and series elastic energy storage (e.g.^29,60^) become available. Still better would be to dispense with the cost coefficient in favor of detailed information about each individual muscle^61,62^, including differences in fiber type and function. We expect improved estimates of elastic contributions, energy transfer, and the cost of performing work to lead to better explanation of the cost of running. However, based on current evidence, it appears that even though series elasticity performs a major role in running, active mechanical work still explains a majority of the metabolic cost in running. Accurate models for estimating this cost are important for understanding human preferences in dynamic activities and can inform the design of devices that interface with the body such as prostheses and orthotics.

## Supplementary Information

Supplementary Information.

## Abbreviations

$M$: Body mass (kg)
$$L$$: Leg length (m)
$$g$$: Gravitational acceleration (m/s^2^)
$c_{ + } ,c_{ - }$: Metabolic costs for positive and negative work respectively (metabolic/mechanical energy, dimensionless)
${c}_{\pm }$: Metabolic cost for net work (metabolic/mechanical energy, dimensionless)
${f}_{\text{M}}$: Muscle work fraction (muscle work divided by total joint work by muscle & tendon, dimensionless)
${W}_{\text{M}}^{+}, {W}_{\text{M}}^{-}$: Muscle positive and negative work, respectively (J)
${W}_{\text{MT}}^{+}, {W}_{\text{MT}}^{-}$: Muscle–tendon positive and negative work, respectively (J)
${W}_{\text{ST}}$: Soft tissue work (J)
${E}_{\text{work}}$: Metabolic energy cost due to mechanical work (J)
SI: Summed Ipsilateral work, an estimate of muscle–tendon work assuming no energy transfer across the pelvis
SB: Summed bilateral work, an underestimate of muscle–tendon work assuming full energy transfer across all joints of the body
IJ: Independent Joint work, an overestimate of muscle–tendon work assuming no energy transfer across all joints of the body
KT cost: The model proposed by Kram and Taylor^1^ to estimate the metabolic cost of generating muscle force
