# Supplementary material for: Mechanical work accounts for most of the energetic cost in human running
Source: Sci Rep. 2022 Jan 12;12:645. doi: 10.1038/s41598-021-04215-6 (PMC8755824; doi:10.1038/s41598-021-04215-6)
Supplement: Supplementary file 1 — Supplementary Information. [file 41598_2021_4215_MOESM1_ESM.docx]

# Supplementary Information

# Title: Mechanical Work Accounts for Most of the Energetic Cost in Human Running

Authors: **Riddick, RC, Kuo, AD**

## Supplementary Appendix S1

Here we use a dimensional analysis to show how the KT cost model^1^ based on body weight and ground contact time could be considered equivalent to a cost of performing work. In fact, it is equivalent to evaluating the present study’s cost of performing muscle work (but only on the centre of mass), using three assumptions: (1) constant speed locomotion, (2) running on level ground, and (3) a single value for the cost coefficient (lumping together the cost of active muscle work and series elastic contribution). The KT model states that metabolic cost is proportional to body weight $Mg$ divided by ground contact time $t_{c}$:

$$\dot{E}=K\cdot Mg/t_{c}$$

Where $K$ is a constant of proportionality. Integrating this equation over stance phase results in the amount of energy used by the body during the stance phase of one step:

$$E=K\cdot Mg$$

Since $E$ has units of energy, $K$ must have units of length. The constant also includes an implicit cost of performing the work, which converts from mechanical to metabolic energy. As we have seen, both the efficiency of muscle performing negative and positive work $c_{\pm}$, and the proportion of work done by muscle $f_{M}$ (as opposed to tendon) are factors in energy cost, and can be summarized using a cost coefficient, the product $f_{M}c_{\pm}$. Expanding the constant of proportionality to include these terms without a loss of generality, the constant $K$ may be considered equivalent to

$$K=f_{M}c_{\pm}\lambda$$

where $\lambda$ is a parameter with units of length since the cost coefficient $f_{M}c_{\pm}$ is dimensionless. Updating and rearranging the equation for the energy cost of a step results in

$$E=f_{M}c_{\pm}\cdot Mg\cdot\lambda$$

The first term $f_{M}c_{\pm}$ is the cost coefficient, the second term is the average load the ground applies on the body $Mg$, and the third term $\lambda$ is therefore the distance displaced by the load, by the definition of mechanical work. This parameter $\lambda$ represents the distance travelled by the body’s center of mass along the direction of the applied ground force. We calculated this value for the running data used in this paper and found it to be equal to 0.090 ± 0.015 *m*. Examining the value for $K$ reported for humans, it has an average value of 0.262 m across a range of running speeds from 2 to 4 m/s, similar to the running speeds in our dataset. Dividing this value reported for $K$ by the average distance travelled by the body $\lambda$, we get a cost coefficient of $f_{M}c_{\pm}=2.91$, slightly higher than the value 2.41 we used, and within the range of reasonable values for the cost coefficient (Figure 4). This shows that the value of $K$ is a single parameter that represents the effects of three distinct physical phenomena: energetic cost of work, series elasticity, and displacement of the COM along the direction of the ground force.

It appears that the KT model is a special case of the cost of generating mechanical work, under steady state, level ground locomotion. To generalize this equation across terrains, accelerations and decelerations, and types of locomotion would be a matter of separately assessing the amount of distance travelled by the body when performing both negative and positive work, and taking into account the different efficiencies for both types of work. Our cost model (equation 2) provides the framework for doing so, and it applicable even when locomotion is not constant speed nor on level ground.
